# HIV Incidence and Spatial Clustering in a Rural Area of Southern Mozambique

**DOI:** 10.1371/journal.pone.0132053

**Published:** 2015-07-06

**Authors:** Raquel González, Orvalho J. Augusto, Khátia Munguambe, Charlotte Pierrat, Elpidia N. Pedro, Charfudin Sacoor, Elisa De Lazzari, John J. Aponte, Eusébio Macete, Pedro L. Alonso, Clara Menendez, Denise Naniche

**Affiliations:** 1 ISGlobal, Barcelona Ctr. Int. Heath Res. (CRESIB), Hospital Clínic-Universitat de Barcelona, Barcelona, Spain; 2 Centro de Investigação em Saúde da Manhiça (CISM), Maputo, Mozambique; 3 Direcção Nacional de Saúde (DNS), Ministério da Saúde, Maputo, Mozambique; 4 World Health Organization (WHO), Geneva, Switzerland; St. James School of Medicine, ANGUILLA

## Abstract

**Background:**

Monitoring the HIV epidemic in a defined population is critical for planning treatment and preventive strategies. This is especially important in sub-Saharan Africa, which harbours the highest burden of the disease.

**Objective:**

To estimate HIV incidence in adults aged 18-47 years old and to investigate spatial variations of HIV prevalence in Manhiça, a semi-rural area of southern Mozambique.

**Methods:**

Two cross-sectional community-based surveys were conducted in 2010 and 2012 to determine HIV prevalence. Individual participants were randomly selected from the demographic surveillance system in place in the area and voluntary HIV counselling and testing was offered at the household level. HIV incidence was calculated using prevalence estimates from the two sero-surveys. Each participant’s household was geocoded using a global information system. The Spatial Scan Statistics programme was used to identify areas with disproportionate excess in HIV prevalence.

**Results:**

A total of 1511 adults were tested. The estimated HIV prevalence in the community was 39.9% in 2010 and 39.7% in 2012. The overall HIV incidence was 3.6 new infections per 100 person-years at risk (PYAR) [95CI 1.56; 7.88], assuming stable epidemic conditions, and tended to be higher in women (4.9/100 PYAR [95CI 1.74; 11.85]) than in men (3.2/PYAR [95CI 1.36; 9.92]). One cluster with significant excess HIV prevalence was identified at the same geographic location in both surveys. This cluster had an HIV prevalence of 79.0% in 2010 and 52.3% in 2012.

**Conclusions:**

The findings of these first individually-randomised community-HIV sero-surveys conducted in Mozambique reinforce the need to combine HIV incidence estimates and research on micro geographical infection patterns to guide and consolidate effective prevention strategies.

## Introduction

Surveillance of the HIV epidemic is essential in sub-Saharan African (SSA) countries where the highest burden of the disease concentrates, accounting for 69% of the people living with HIV worldwide[1]. HIV prevalence estimates have been based on data from women attending the antenatal clinics and more recently on population-based surveys[2]. However, for monitoring the HIV epidemic prevalence measures may not be as useful as it is measuring the appearance of new infections. The accepted gold standard method for measuring population-level HIV incidence is a prospective cohort study that assess the occurrence of new infections in a well-defined HIV-negative population followed over time and tested at regular intervals for HIV infection[3, 4]. However, this method is logistically complex and costly. Over the last years, methods for estimating HIV incidence using data from single-round cross-sectional surveys have been developed and validated in several countries[3–7].

While the HIV epidemic has been extensively studied through prevalence and incidence estimates, geographic patterns of HIV distribution at local community level have been underexplored in SSA. The analysis of HIV epidemics at a micro geographic scale including identification of hot spots is necessary to understand the driving forces of HIV transmission patterns [8]. Spatial analysis at the local level may also enable policy makers to design effective and culturally acceptable preventive measures or outreach programs for increasing antiretroviral therapy (ART) uptake in clusters of high HIV prevalence.

Mozambique is one of the ten countries with the highest HIV prevalence in the world with 1.4 million (95% Confidence Interval (95CI) 1.2–1.5) people living with HIV according to UNAIDS estimates [9, 10]. The first population-based survey conducted in 2009 showed that national HIV prevalence was 15% (95CI 13.9–16) in individuals aged 15–49 years old reaching over 19% in the southern region of the country[11]. Recent community-based estimates from central and southern Mozambique indicate that HIV prevalence may be up to 32.6% and 39.9%, respectively in some populations[12, 13]. No data on HIV incidence at community-level are currently available.

The present study reports the results of two community based cross-sectionals surveys to estimate HIV prevalence and incidence in adults aged 18–47 years in a semi-rural district of southern Mozambique. In addition, a geographical analysis was performed in order to investigate spatial variations and clusters of HIV prevalence in the area under study.

## Methods

### Ethics statement

The study protocol and informed consent form were reviewed and approved by the National Committee on Health Bioethics of Mozambique and the Hospital Clínic of Barcelona Ethics Committee (Spain). Written informed consent was obtained from all study participants.

### Study area and population

The study was carried out in Manhiça District, a semi-rural area in Maputo Province, in southern Mozambique. The characteristics of the study population and site have been described elsewhere [12, 14]. Briefly, since 1996 the Centro de Investigação em Saúde de Manhiça (CISM) runs a continuous demographic surveillance system (DSS) for vital events and migrations. In 2007, there were 160 000 inhabitants in the district and currently the DSS covers over 92 000 inhabitants [15]. The preferred destinations for emigrants from Manhiça are the Maputo city followed by South Africa and other districts within Maputo province[14]. Since 2003 the CISM collaborates with the Mozambican HIV/AIDS control program through the establishment and continuous support of voluntary counseling and testing centres at health facilities, provision of antiretroviral drugs and diagnosis tests, and contributing to the clinical management of patients among other activities. Estimates from 2010 showed that the HIV prevalence at the antenatal clinics of the Manhiça District Hospital was 29.4%, and of 39.9% at the community level [12].

### Study design

Two cross sectional community-based studies to determine age and sex-specific HIV prevalence in individuals aged 18–47 years old were conducted in 2010 [12] and 2012. The sample size was calculated with a precision of 0.05 assuming a 20% HIV prevalence and a 95% confidence interval and it was deemed that 232 subjects per age group would be needed to determine age and sex specific prevalence. The width of the defined age groups was designed to be equal among the three age groups (18–27, 28–37 and 38–47 years of age). A secondary objective was to calculate HIV incidence in the community from cross-sectional prevalence data at two pre-defined time points [3, 5–7]. In order to ensure HIV incidence calculations for all age groups, the second cross sectional survey included an upper age group of 47–50 since the model takes into account the time interval between surveys[6].

### Study procedures

Selection of participants has been described in detail elsewhere [12]. Briefly, random lists of adults living in the study area stratified by age group and sex were generated and organized by neighborhood. The study inclusion criteria were: age 18–47 or 18–50 years in the first and second surveys respectively, being resident of the main study area, and willing to participate in the study after signing a written informed consent. The individuals were visited at home by a study field worker who explained the objectives of the study. If the candidate agreed, another home visit was made to provide more information about the study and HIV testing. Voluntary HIV counselling and testing was offered at the household level but only the randomly selected individuals contributed data to prevalence estimates. Study procedures of the household visits have been described in detail elsewhere[12]. Recruitment was stopped once the minimum sample size for each age and sex group was reached. Basic sociodemographic information was recorded onto a specifically designed case report form using mobile devices. Rapid HIV testing was performed by fingerprick following national recommendations using two rapid tests; the Determine HIV 1/2 test (Abbott laboratories, North Chicago, IL, USA), and the UniGold HIV test for confirmation of an HIV-positive result (Trinity Biotech, Bray, Ireland). HIV positivity was defined only if both tests were positive. Participants with a positive result were offered medical follow up at the Manhiça outpatient clinic, which included CD4 counts, clinical management and provision of ART according to national guidelines [16].

### Data management and statistical analysis

Data from the mobile devices was transferred to the CISM centralized database. The Open Data Kit software (http://opendatakit.org/) was used for data management. Information on participant’s religion and migration history was obtained from the CISM DSS [17]. Individuals under the DSS are considered immigrants if they were not born in the Manhiça DSS area and had lived there for at least three months [14]. One-way and two-way contingency tables were generated for description of the categorical variables and calculation of proportions and p-values, taking into account the probability of sampling and stratification to extrapolate the data from the survey to the community[18]. The statistical analysis was performed using Stata statistical software version 12 (Stata Corp., College Station, Texas, USA).

### HIV incidence estimation

HIV incidence was derived from the HIV prevalence estimates at the two cross-sectional surveys from 2010 and 2012 according to the model proposed by Hallet *et al*.[6, 19, 20] and validated in other settings [4, 5]. The method has been described in detail elsewhere [5, 6]. Briefly, it is based on the synthetic cohort principle and relies on the decomposition of prevalence changes by age group of width *r* (usually 5 years) between two cross-sectional surveys separated by *T* years of time. It assumes that individuals of age *a* years in the first survey will be represented by individuals aged *a* + *T* years in the second survey. Thus, the HIV prevalence in the second of two surveys represents the sum of the incident HIV infections and the surviving HIV-infected individuals from the first survey, even if the survey does not include the same individuals. The model adjusts for mortality rates using assumptions for three HIV epidemic situations, early, stable and declining [5, 6, 21]. Confidence intervals of incidence estimates were generated by bootstrap [19]. HIV incidence was estimated by age groups of 5 years width.

### Spatial clustering detection

For the spatial analysis, “clusters of HIV infection” were defined as “geographical areas with a disproportionate excess in HIV prevalence compared to the surrounding areas”[22]. In a specific scale, the importance of HIV infection of an area defined as a cluster differs from that of a neighborhood area. Spatial Scan Statistics (SaTScan) programme (http://www.satscan.org/) and Kulldorf’s methodology were used to identify clusters of HIV infection [22–24]. For detecting clusters, SaTScan gradually scans a window across space and compares the number of observed and expected cases inside the window at each location. The radius of the window varies continuously in size from zero to an upper limit of 50% of the entire study area. This method creates an infinite number of circular windows [24]. The window with the maximum likelihood ratio is identified as the “most likely cluster”. For each window, the likelihood function is computed as follows:

$${\left(\frac{c}{E\left[c\right]}\right)}^{c}{\left(\frac{C-c}{C-E\left[c\right]}\right)}^{C-c}$$

Where *C* is the total number of cases, *c* is the observed number of cases within the window and *E*[*c*] is the covariate adjusted expected number of cases within the window under the null hypothesis. SaTScan takes into account and adjusts for population density in the analysis. Finally, Monte Carlo hypothesis testing was performed to calculate p-values for detected clusters. The number of DSS individuals that were eligible for participation at the first and second survey (background population at risk) was taken into account for cluster identification (N = 13618 and N = 20309, respectively).

## Results

### HIV prevalence in 2010 and 2012 surveys

**Fig 1:**
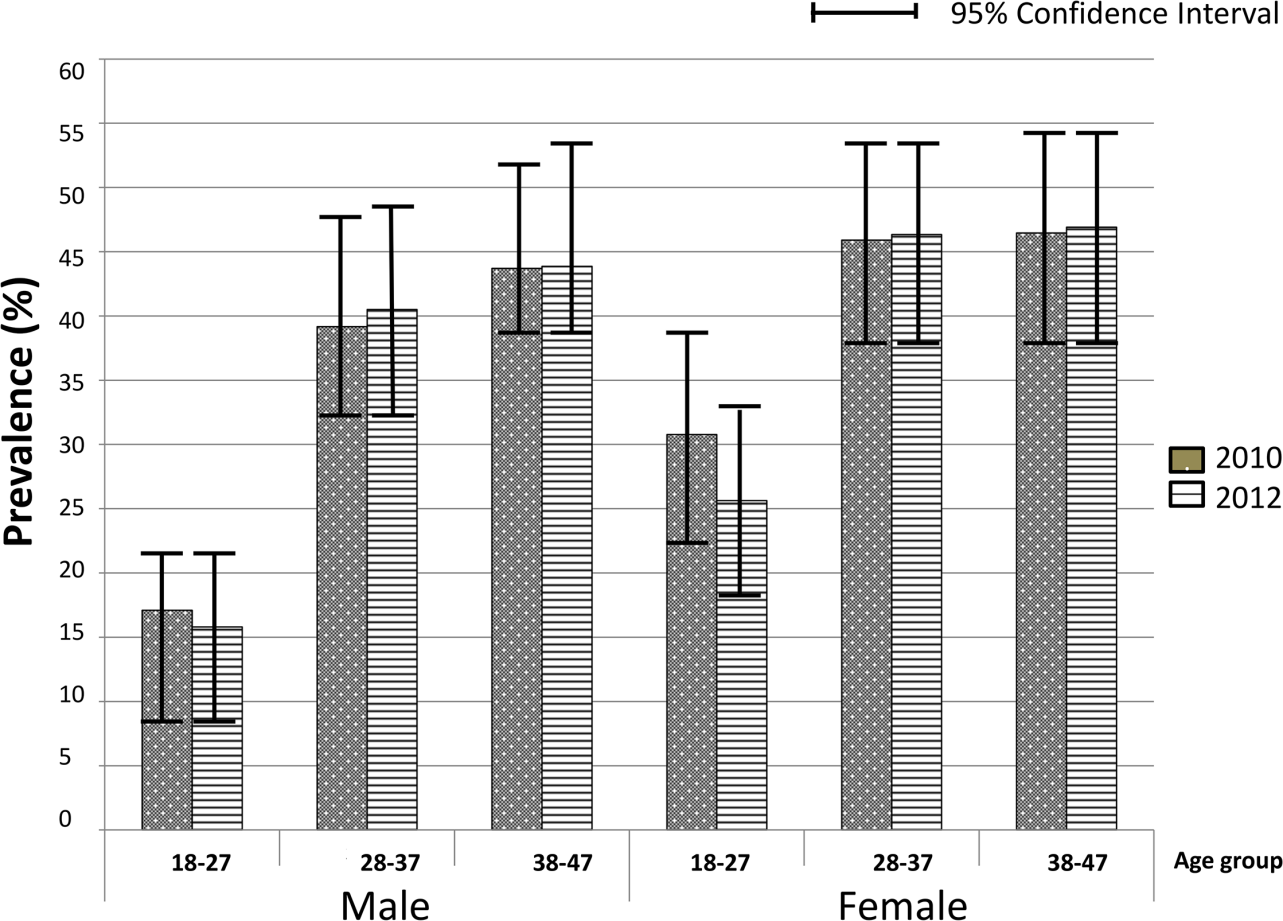
Age and sex specific HIV prevalence in 2010 and 2012.

Detailed results of the 2010 sero-survey are described in Gonzalez *et al.[12]*. In 2012, 888 adults were approached and given an appointment card for a later mobile team visit, following the same methodology as the previous survey [12]. Of the 888 adults invited, 789 accepted to participate and were recruited with an acceptance rate of 88.9%, similar to that of 2010 of 86.1%[12]. There were no significant differences in sex, age or education of participants between surveys (Table 1). The community HIV prevalence in 2012 was 39.7% (95CI 36.0;43.5), similar to the 39.9% (95CI 35.9;43.8) observed in 2010[12]. The age and sex-specific HIV prevalence were also similar in both surveys (Fig 1). HIV prevalence was significantly lower in the 18–27 year age group for both men and women as compared to the older age groups in both survey years (p<0.001).

**Table 1:** Socio-demographic characteristics of study participants in both HIV sero-prevalence surveys

		2010*		2012	
		n	%	n	%
**Sex**					
	**Male**	356	49.3	384	48.7
	**Female**	366	50.7	405	51.3
**Age group**					
	**18–27**	234	32.4	235	29.8
	**28–37**	242	33.5	244	30.9
	**38–47**	246	34.1	227	28.7
	**48–50**	—	—-	83	10.5
**Education**					
	**No schooling**	103	14.4	160	20.3
	**Primary school**	422	58.9	388	49.2
	**Secondary school**	172	24.0	218	27.6
	**Superior**	19	2.7	23	2.6

* data from Gonzalez *et al*. 2012 [12]

### HIV incidence estimates

Indirect HIV incidence estimates derived from HIV prevalence approximated 2.9 new infections/100 person-year (PYAR) (95CI 1.40; 7.14) for early epidemic conditions, 3.6 infections/100 PYAR (95CI 1.56; 7.88) for a stable HIV epidemic, and 4.7 infections/100 PYAR (95CI 2.37; 8.99) for declining epidemic conditions. HIV incidence point estimates in the three epidemic conditions tended to be higher in women (stable epidemic: 4.9/100 PYAR [95CI 1.74; 11.85]) than in men (stable epidemic: 3.2/PYAR [95CI 1.36; 9.92]; Table 2). Age stratification by 5 years age-group revealed a peak in HIV incidence trends at 35–39 years of age which was not statistically significant (S1 Fig).

**Table 2:** HIV incidence estimates assuming three different HIV epidemic conditions

	HIV epidemic					
	Early		Stable		Declining	
	Incidence*	95%CI	Incidence*	95%CI	Incidence*	95%CI
**Overall**	2.892	1.401; 7.136	3.620	1.559; 7.878	4.731	2.370; 8.993
**Males**	2.754	1.128; 9.389	3.211	1.355; 9.917	4.117	1.928; 10.924
**Females**	4.342	1.628; 11.023	4.916	1.744; 11.852	6.063	2.583; 13.053

* New infections per 100 person-years at risk (PYAR)

### Spatial analysis of HIV prevalence

**Fig 2:**
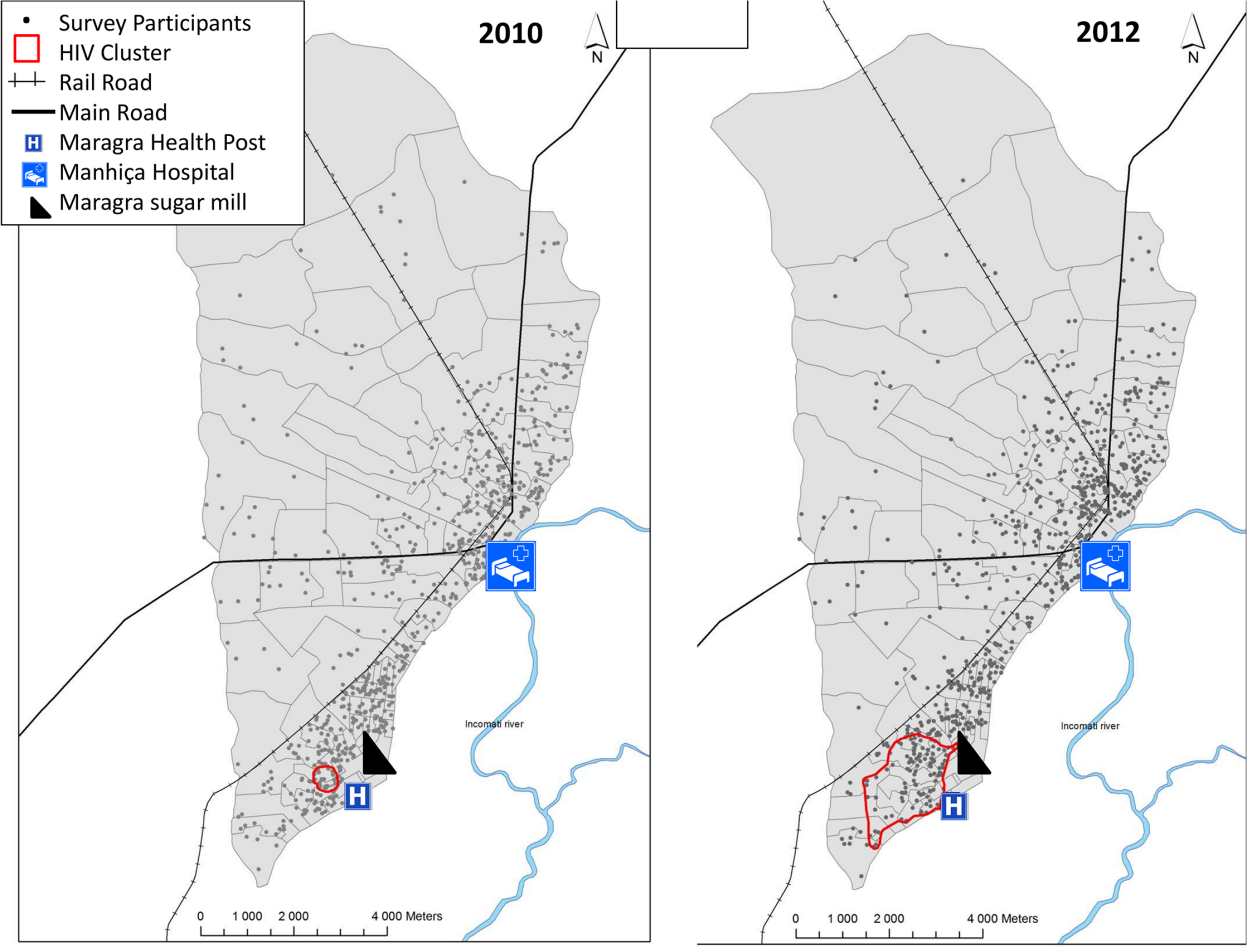
Maps of the 2010 and 2012 Spatial Analysis identifying HIV clusters in Manhiça district.

The spatial scan analysis identified a small cluster of significant excess of HIV prevalence in 2010 (<0.05) with 19 individuals out of the 722 included in the analysis, and an overall area of 0.2 kilometres^2^ (km^2^) (Fig 2). HIV prevalence within this cluster was 79.0% (*versus* 36.5% outside). When using data from the 2012 sero-survey, the spatial scan identified a cluster of higher HIV prevalence centred in the same location as in 2010 but spanning a larger geographical area with 109 individuals out of the 789 included in the analysis (Fig 2). The 2012 cluster was approximately 1.4 km from the Maragra sugar mill, had an overall perimeter of 7.6 km and spanned 2.7 km^2^. The HIV prevalence inside the 2012 cluster was 52.3% (95CI 42.9; 61.7) as compared to 34.7% (95CI 31.1; 38.3) outside (p = 0.002; OR = 2.05 [95CI 1.36; 3.08]; p = 0.001). S2 Fig shows a map of the Manhiça DSS population density in 2012 and location of the cluster, which is independent of population density. There were no significant differences in sex, age, marital status education orliteracy between individuals from the high HIV prevalence cluster and those from the lower prevalence surrounding area (Table 3). There was however a significant difference in the proportion of immigrants within the cluster (56.9%) compared to that outside (39.6%; p = 0.001).

**Table 3:** Characteristics of the 2012 HIV cluster population as compared with those residing outside of the cluster.

		ClusterN = 109		Outside of cluster N = 680		p-value
		n*	%*	n*	%*	
**HIV cases**		57	52.3	236	34.7	**0.002**
**Sex**						
	**Male**	59	54.1	325	47.8	0.219
**Age** *(mean*, *SD)*		34.3	0.87	34.4	0.37	0.985
**Marital status**						0.892
	**Married**	5	4.6	28	4.1	
	**Living with partner**	76	69.7	444	65.3	
	**Single**	22	20.2	160	23.5	
	**Divorced/Widowed**	6	5.5	48	7.1	
**Education**						0.175
	**No schooling**	2	2.3	6	1.1	
	**Primary school**	59	68.6	329	59.7	
	**Secondary school**	21	24.4	197	35.8	
	**Superior**	4	4.7	19	3.5	
**Literacy**						
	**Yes**	86	78.9	551	81.0	0.601
**Immigrants**						
	**Yes**	62	56.9	269	39.6	**0.001**
**Religion**						0.190
	**Christian**	66	61.1	443	67.5	
	**Other**	42	38.9	213	32.5	

* Unless otherwise specified; p-value from Chi^2^.

## Discussion

This study has shown that in the DSS area of Manhiça, in southern Mozambique, the average adult community HIV prevalence in 2012 was very high, nearly 40%, with little change over that found two years earlier. Prevalence was highest in the older population as compared to the 18–27 age group. The estimated number of new infections was 3.6/ 100PYAR, with a slightly higher rate in women at 4.9 /100 PYAR. The findings also show that there is spatial heterogeneity in the observed HIV prevalence within the Manhiça DSS, with a small cluster (of 0.2 km^2^ area) of significant excess of HIV prevalence identified in the 2010 survey which, increased to a 2.7 km^2^ area in the 2012 survey.

The consistent results showing high prevalence of HIV both in 2010 and 2012 in Manhiça confirms the magnitude of the epidemic in this southern region of the country. A proportion of the high prevalence is likely to be due to increased survival in HIV-positive individuals stemming from the scale up of ART coverage. High HIV prevalence has been described in young women from the centre of Mozambique and in neighbouring countries such as South Africa and it has been associated with both continued new infections, as well as increased survival of the HIV-positive population receiving ART [13, 25–28]. The impact of ARTs rollout on contributing to increased HIV prevalence has recently been shown in neighbouring KwaZulu Natal, South Africa. This was found to be the greatest at the initial phases of the ART scale up, from zero to 40% coverage (between 2004 and 2010), stabilizing afterwards despite continued increases in ART coverage [26, 29]. Mozambique, like KwaZulu Natal, began ART scale-up in 2003 and recent reports estimate that ART coverage of adults in need of treatment in Mozambique has hovered at an estimated 50% since 2010 [30, 31]. This level of ART coverage is indeed associated with decreased risk of HIV acquisition [32], however there still remains a substantial pool of HIV-infected untreated individuals potentially contributing to the propagation of new infections, with the incidence of new infections suggested to be higher in the older population [29, 33].

In this study, an overall crude incidence of 3.6 new HIV infections/100 PYAR was estimated from prevalence sero-surveys in Manhiça, assuming stable epidemic conditions. Sex-specific incidence was estimated at 4.9 infections/100 PYAR for women and 3.2 infections/100 PYAR for men Previous estimates of HIV incidence in Manhiça from prevalence data in women of reproductive age from 1999 to 2008 ranged from 11.6 to 12.2 infections/100 PYAR[19]. Although study populations are not comparable, the current data points to a possible decrease in incidence of new infections in this area of the country. In neighbouring KwaZulu Natal estimated HIV incidence has been reported from longitudinal cohorts with a range of 6.3 to 14.8 new infections per 100 PYAR in 2009 and timid decreases in new infections since 2011 [25, 34]. Two recent cohort studies conducted among women at higher risk for HIV acquisition in neighbouring Mozambican areas report similar incidences to that found in Manhiça, of 4.6 infections/100 PYAR (95CI 2.7; 7.3) and 6.5 infections/ 100 PYAR (95CI 4.1; 9.9)[28, 35]. It has been described that HIV incidence may be lower in younger population[29]. However, the sample size in this study did not allow for precise estimations of incidence by age groups. The age-specific incidence estimates from the Manhiça population suggests trends of higher incidence at 35–40 years of age, in agreement with analyses using household-based prevalence data from Zambia and Niger that showed a peak of HIV incidence in men of the same age group[5]. In Manhiça, this peak was observed for the estimated incidence and it was not seen for age-specific HIV prevalence. It has been suggested that HIV incidence at older ages is due to widowhood exposing individuals to the risk of infection as they form new partnerships[36]. Apart from that, it has been described that peak HIV incidence in men may occur 5–7 years later than in women in a mature epidemic [5, 27].

In addition to population-based prevalence and incidence measures, the estimation of geographic distribution of HIV infection is essential in developing strategies for tackling the epidemic. These results suggest a significant geospatial cluster of excess HIV prevalence in a 7.6 km perimeter area within the Manhiça DSS, which is near to the Maragra sugar mill. Indeed, in 2012 HIV prevalence was 52.3% within what it can be considered a “hot spot”, as compared to 34.7% outside (p = 0.002). This cluster near the sugar mill was not associated with demographic risk factors for HIV except a higher rate of immigration. Southern Mozambique, similar to KwaZulu Natal in South Africa, is a stronghold of sugar production. Mozambique has four sugar mills, two of which, including Maragra, are in the Maputo province. Although sugar mills employ permanent workers, much of the workforce in the sugar industry rests on hired seasonal cane cutters. In Maragra, of a total workforce of approximately 5500 in 2012, close to 3000 were seasonal workers [37]. Studies in KwaZulu Natal and Malawi have described that migrants and their partners are at greater risk for HIV and other STIs [38]. This may be associated with increased frequency of sexual relationships outside of the couple and the effect of concurrent relationships with split households [39, 40]. The mobility of sugar mill workers is likely to be associated with a higher cluster of HIV prevalence. The occurrence of micro geographic clustering of HIV prevalence was recently described in a study across SSA using national demographic data[8]. In Mozambique, several high HIV prevalence clusters were found in Maputo, Gaza, Sofala and Zambezia provinces using data from 2009 [8]. Indeed, comparable to what observed in the Manhiça DSS area, micro clusters of high prevalence have also been observed at the level of neighbourhoods in a Tanzanian study conducted with HIV AIDS indicator survey data from 2003–2004[41]. It is challenging to assess the contribution of different risk factors to micro geographic clusters in HIV due to the sheer number of potential risk factors and the small population sizes in clusters. Small numbers also preclude accurate estimates of incidence within the clusters. Cuadros *et al*. have proposed that clustering may reflect differences in specific behavioural and biological factors which become amplified as a consequence of the epidemic being close to its epidemic threshold in SSA, thus leading to pockets of higher or lower HIV prevalence [8].

A possible limitation of the study could be that the HIV-specific mortality rates used in the estimates were derived from the model described by Hallett *et al* [5] and not specific to Manhiça. As an example, if the mortality rate used in our methodology was lower than that of Manhiça, HIV incidence would have been overestimated. However, small changes in mortality rates in the methodology do not significantly affect the incidence estimates. Furthermore, this methodology has been validated and employed successfully in many other SSA epidemiological settings [4, 19].

Most HIV prevention programs are guided by HIV prevalence data from administrative districts and HIV incidence data from specific risk groups. Results from Manhiça and other areas in SSA suggest the importance of taking into account “place” in terms of local geospatial differences in HIV prevalence in order to better understand the epidemic[38, 41, 42]. Geospatial prevalence data cannot be used alone since it does not estimate the rate of new infections. It is thus important to combine incidence estimates of new infections with geospatial estimates of prevalence clusters in order to best tailor local interventions and outreach programs for increasing ART uptake and retention in the complex SSA setting.

In conclusion, this study has shown that in this area of southern Mozambique the elevated HIV adult community prevalence remains highest in the older population and is accompanied by a continued high number of new infections especially among women. In addition, a geographic “hot spot” has been identified possibly associated with immigration to the area. These findings reinforce the need for continuous monitoring of the HIV epidemic and for further research on the geographical patterns of the infection, in order to guide and consolidate effective prevention strategies.

## Supporting Information

S1 Fig HIV incidence by age group and HIV epidemic condition. (TIF)

S2 Fig Population density of the study area in 2010 and 2012 with the HIV clusters designated. (TIF)

S1 Table Mortality rates and HIV prevalence estimates by age group and epidemic condition. (DOCX)
