# Supplementary figures and images for: HIV Incidence and Spatial Clustering in a Rural Area of Southern Mozambique
Source: PLoS One. 2015 Jul 6;10(7):e0132053. doi: 10.1371/journal.pone.0132053 (PMC4493140; doi:10.1371/journal.pone.0132053)

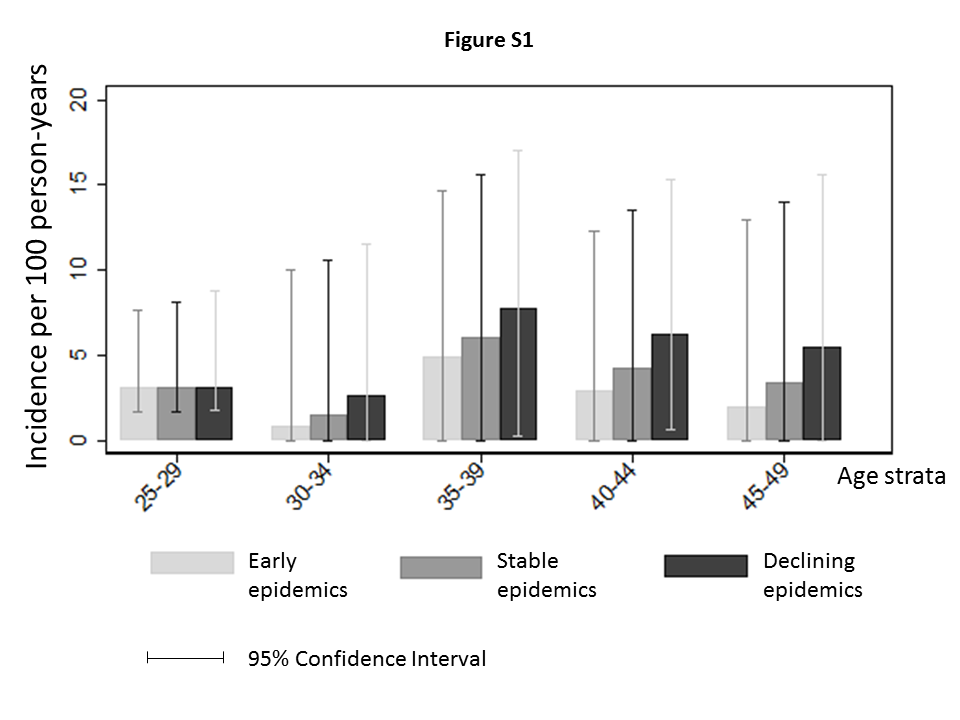

Supplement: S1 Fig — (TIF) [file pone.0132053.s001.tif]

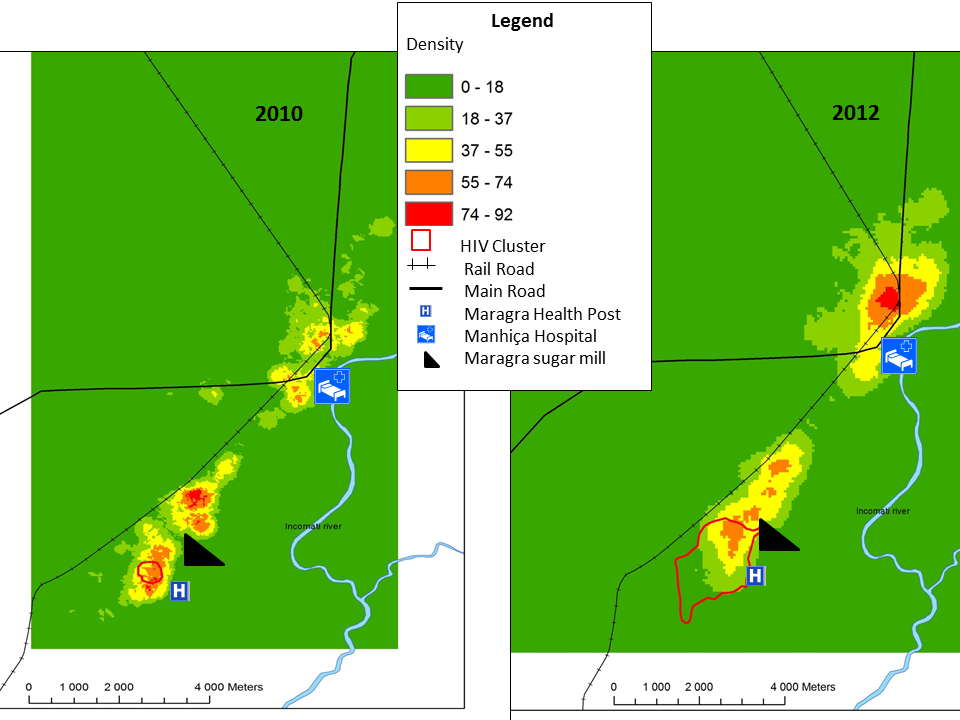

Supplement: S2 Fig — (TIF) [file pone.0132053.s002.tif]
