# Supplementary material for: HIV Incidence and Spatial Clustering in a Rural Area of Southern Mozambique
Source: PLoS One. 2015 Jul 6;10(7):e0132053. doi: 10.1371/journal.pone.0132053 (PMC4493140; doi:10.1371/journal.pone.0132053)
Supplement: S1 Table — (DOCX) [file pone.0132053.s003.docx]

**Supplementary Table S1**

**Mortality rates and HIV prevalence estimates by age group and epidemic condition**

| **Early**  **Epidemic** | **Age- group** | **Mortality rate** | | | **Prevalence (n)** | |
| --- | --- | --- | --- | --- | --- | --- |
|  |  | **HIV+** | | **HIV-** | **2010** | **2012** |
|  | 25-29 | 0.029 | | 0.01 | 42.55 (99) | 37.31 (120) |
|  | 30-34 | 0.036 | | 0.01 | 40.74 (114) | 40.75 (111) |
|  | 35-39 | 0.043 | | 0.01 | 45.55 (144) | 50.23 (141) |
|  | 40-44 | 0.051 | | 0.01 | 45.05 (129) | 45.47 (145) |
|  | 45-49 | 0.058 | | 0.01 | 41.51 (54) | 41.53 (98) |
| **Stable Epidemic** | **Age-group** | **Mortality rate** | | | **Prevalence (n)** | |
|  |  | **HIV+** | **HIV-** | | **2010** | **2012** |
|  | 25-29 | 0.048 | 0.01 | | 42.55 (99) | 37.31 (120) |
|  | 30-34 | 0.059 | 0.01 | | 40.74 (114) | 40.75 (111) |
|  | 35-39 | 0.07 | 0.01 | | 45.55 (144) | 50.23 (141) |
|  | 40-44 | 0.083 | 0.01 | | 45.05 (129) | 45.47 (145) |
|  | 45-49 | 0.095 | 0.01 | | 41.51 (54) | 41.53 (98) |
| **Declining epidemic** | **Age-group** | **Mortality rate Prevalence (n)** | | | | |
|  |  | **HIV+** | **HIV-** | | **2010** | **2012** |
|  | 25-29 | 0.077 | 0.01 | | 42.55 (99) | 37.31 (120) |
|  | 30-34 | 0.095 | 0.01 | | 40.74 (114) | 40.75 (111) |
|  | 35-39 | 0.114 | 0.01 | | 45.55 (144) | 50.23 (141) |
|  | 40-44 | 0.134 | 0.01 | | 45.05 (129) | 45.47 (145) |
|  | 45-49 | 0.154 | 0.01 | | 41.51 (54) | 41.53 (98) |
